# Liposomes Co-Encapsulating Cisplatin/Mifepristone Improve the Effect on Cervical Cancer: In Vitro and In Vivo Assessment

**DOI:** 10.3390/pharmaceutics12090897

**Published:** 2020-09-22

**Authors:** Fabricio Ledezma-Gallegos, Rafael Jurado, Roser Mir, Luis Alberto Medina, Laura Mondragon-Fuentes, Patricia Garcia-Lopez

**Affiliations:** 1 Laboratorio de Farmacologia, Subdirección de Investigación Básica, Instituto Nacional de Cancerología, Cd. México 14080, Mexico; faledezmag@yahoo.com.mx (F.L.-G.); f.rafael.jurado@gmail.com (R.J.); roser.mir@grifols.com (R.M.); 2 Posgrado en Ciencias Biológicas, Universidad Nacional Autónoma de México, Coyacán, Cd. México 04510, Mexico; 3 Unidad de Investigación Biomédica en Cáncer INCan-UNAM, Instituto Nacional de Cancerología, Cd. México 14080, Mexico; medina@fisica.unam.mx (L.A.M); l.mondragon@clinstile.com (L.M.-F.); 4 Instituto de Física, Universidad Nacional Autónoma de México, Coyoacán, Cd. México 04510, Mexico

**Keywords:** cervical cancer, cisplatin, co-encapsulation, drug combination, liposomes, mifepristone, synergism

## Abstract

Cervical cancer is usually diagnosed in the later stages despite many campaigns for early detection and continues to be a major public health problem. The standard treatment is cisplatin-based chemotherapy plus radiotherapy, but patient response is far from ideal. In the research for new drugs that enhance the activity of cisplatin, different therapeutic agents have been tested, among them the antiprogestin mifepristone. Nevertheless, the efficacy of cisplatin is limited by its low specificity for tumor tissue, which causes severe side effects. Additionally, cervical tumors often become drug resistant. These problems could possibly be addressed by the use of liposome nanoparticles to encapsulate drugs and deliver them to the target. The aim of this study was to prepare liposome nanoparticles that co-encapsulate cisplatin and mifepristone, evaluate their cytotoxicity against HeLa cells and in vivo with subcutaneous inoculations of xenografts in nu/nu mice, and examine some plausible mechanisms of action. The liposomes were elaborated by the reverse-phase method and characterized by physicochemical tests. The nanoparticles had a mean particle size of 109 ± 5.4 nm and a Zeta potential of −38.7 ± 1.2 mV, the latter parameter indicating a stable formulation. These drug-loaded liposomes significantly decreased cell viability in vitro and tumor size in vivo, without generating systemic toxicity in the animals. There was evidence of cell cycle arrest and increased apoptosis. The promising results with the co-encapsulation of cisplatin/mifepristone warrant further research.

## 1. Introduction

Cervical cancer is the fourth most common malignant neoplasm and the fourth leading cause of cancer death in women worldwide [1], being especially problematic in developing countries such as Mexico [2]. Since it is initially a slow-growing cancer, there are many campaigns for early detection and tests are widely available. Nevertheless, it is often detected in the later stages when rapid proliferation is underway. The conventional treatment given is surgery followed by cisplatin-based chemotherapy plus radiotherapy [3]. The introduction of chemo-radiation in 1999 (versus radiation alone) led to improvements in survival in the early stages of cervical carcinoma [4]. However, the prognosis for patients in stages III and IV is still unfavorable [5,6].

One of the principal disadvantages of cisplatin and other types of chemotherapy is their lack of specificity, leading to dose-dependent damage to normal tissues. As a result, patients have low tolerability to treatments and a poor quality of life. The severe adverse effects of cisplatin include nephrotoxicity, peripheral neuropathy, myelotoxicity and ototoxicity [7], in part caused by its poor pharmacokinetic behavior. More than 90% of the drug becomes irreversibly inactivated by binding to proteins, thus leaving only a small percentage of the dose for cytotoxic activity at the site of the tumor [8]. In addition to its side effects, another disadvantage of cisplatin treatment is the rapid development of tumor resistance [9].

Consequently, new methods are constantly being sought to enhance patient response to cervical cancer therapy. One focus of research is to design and develop new analogues of cisplatin. For example, carboplatin and oxaliplatin were each able to slightly decrease side effects, but their efficacy was not better. Moreover, other anti-tumor drugs, including paclitaxel, 5-fluorouracil and monoclonal antibodies, have been combined with the standard cisplatin chemotherapy. The resulting therapeutic efficacy is controversial and in several cases the patients experience greater toxicity [10].

Another approach for improving cervical cancer treatment is to seek chemo-sensitizing agents capable of enhancing the efficacy of cisplatin, such as the antiprogestin mifepristone. This synthetic steroid is used as an abortifacient drug because of its anti-progestational and anti-glucocorticoid action. Our group previously showed during a preclinical trial that there are better outcomes of chemo-radiotherapy with the addition of mifepristone (RU486) [11,12], and demonstrated synergism between mifepristone and cisplatin to act against the proliferation of cervical cancer cell lines in vitro and cervical cancer xenografts in vivo [12,13].

Although cervical cancer is traditionally considered unresponsive to antihormonal therapy [14], the mechanisms of action of mifepristone described in the literature seem to hold promise for facilitating the response to chemo-radiation in non-hormone dependent tumors. One of the mechanisms found was a mifepristone-induced decrease in the vascular endothelial growth factor (VEGF) expression in cervical xenografts [12]. Hence, VEGF down-regulation may be one of the mechanisms involved in the combined mifepristone-cisplatin activity. Several authors have demonstrated that mifepristone can serve as a chemosensitizing drug in combination with cisplatin in non-small cell lung carcinoma, ovarian, endometrium and colorectal cancer [15,16,17,18].

Cervical cancer therapy remains far from ideal in spite of the addition of mifepristone, particularly because of the lack of specificity of cisplatin, leading to the difficulty of accumulating the drug at the site of the tumor. An attractive therapeutic option to address these problems is the development of drug delivery systems, such as nanosystems, to improve the efficacy, safety, physicochemical properties and pharmacokinetic/pharmacodynamic profiles of anti-cancer agents [19,20]. One example is the use of drug-loaded liposomes to transport active molecules to the desired target, thus decreasing systemic toxicity and increasing bioavailability. The active substances are embedded in the lipid bilayer or encapsulated in the aqueous interior of liposomes.

According to several studies, liposomes are rapidly recognized and removed from circulation by the mononuclear phagocyte system (MPS) and the reticuloendothelial system (RES) [21]. However, if the liposome carrier system is coated with the polymer polyethylene glycol (PEG), it acquires the important characteristic of prolonged circulation in vivo, implying a substantial increase in drug bioavailability by the steric stabilization of the nanosystem [22,23]. Although various liposomal formulations of cisplatin have been investigated in recent years, there are few reports on the co-encapsulation of two anticancer drugs in a nanosystem.

The aim of the present study was to prepare liposome nanoparticles that co-encapsulate cisplatin and mifepristone, evaluate their cytotoxicity against HeLa cells in vitro and HeLa cell xenografts in nu/nu mice, and examine possible mechanisms as an analysis of cell cycle and apoptosis at different times. The liposomes were elaborated by the reverse-phase method and characterized by physicochemical tests.

## 2. Materials and Methods

### 2.1. Drugs and Chemicals

Cisplatin, mifepristone, trypsin, sodium chloride, nickel chloride and sodium diethyldithiocarbamate were obtained from Sigma-Aldrich Chemical Co. (St. Louis, MO, USA). Hydrogenated soybean l-α-phosphatidylcholine (HSPC, Avanti Polar Lipids, Inc., Alabaster, AL, USA, 1,2-distearoyl-sn-glycero-3-phosphoethanolamine-N [methoxy (polyethyleneglycol)-2000] (DSPE-mPEG2000, Avanti Polar Lipids Inc., Alabaster, AL, USA) and cholesterol (Avanti Polar Lipids Inc., Alabaster, AL, USA). HPLC-grade acetonitrile, chloroform and methanol were acquired from Honeywell International, Inc. (Morristown, NJ, USA). Dulbecco’s modified Eagle’s medium (DMEM, Thermo Fisher Scientific Inc., Waltham, MA, USA), fetal calf serum (FCS, Thermo Fisher Scientific Inc., Waltham, MA, USA), ethylenediaminetetraacetic acid (EDTA, Thermo Fisher Scientific Inc., Waltham, MA, USA), Tris and SDS were procured from GIBCO Inc. (Grand Island Biological Company, New York, NY, USA). High-quality water employed to prepare solutions was provided by Milli-Q Reagent Water System Continental Water Systems (Millipore, El Paso, TX, USA).

### 2.2. Preparation of Cisplatin/Mifepristone-Loaded Liposomes

Liposomes containing both cisplatin and mifepristone (L-Cis/MF) were elaborated based on a modified version of reverse-phase evaporation [24]. Briefly, a mixture of HSPC, cholesterol and DSPE-mPEG_2000_ at a molar ratio of 60:35:5 was dissolved in chloroform/methanol (2:1 *v*/*v*, final volume 6 mL/batch) and mixed with 5 mg of mifepristone. This mixture was slowly added dropwise into a saturated cisplatin solution in sterile water (8 mg/mL) heated at 65 °C, always maintaining a molar ratio of cisplatin/phospholipids of 1:12. The organic solvents were removed in a rotatory evaporator and the suspension was sonicated for 2 h to reduce and homogenize the liposome size. Cisplatin liposomes (L-Cis) were prepared in the same way but without mifepristone in the lipid solution. Non-encapsulated cisplatin was removed by dialysis at room temperature for 4 h in saline solution using a 12,000 Da MWCO membrane (Spectrum Labs Inc., San Francisco, CA, USA), while non-encapsulated mifepristone was separated by molecular exclusion chromatography on Sephadex-G-25 in PD-10 columns (GE Healthcare Inc., Chicago, IL, USA), eluting and collecting 40 fractions (equilibrated with PBS, pH 7.4). Empty liposomes (L-Control) were prepared in the same way but without adding any drug. The liposomes were stored at 4 °C and protected from light.

### 2.3. Characterization of Cisplatin/Mifepristone-Loaded Liposomes

The mean particle size, polydispersity index (PDI) and Zeta potential of L-Cis and L-Cis/MF were measured by the dynamic light scattering (DLS) technique on a 90Plus Particle Size Analyzer (Brookhaven Instruments Corporation, Holtsville, NY, USA) at 25 °C. The phospholipid content was quantified by the ammonium ferrothiocyanate method [25]. Briefly, an aliquot of the liposomes (L-Cis or L-Cis/MF) was dried and mixed with chloroform before adding an equal volume of ammonium ferrothiocyanate. The mixture was shaken vigorously, the samples were centrifuged, and then the absorbance of the chloroform layer was read at 488 nm in a DU^®^ 530 spectrophotometer (Beckman Coulter, Brea, CA, USA). The calibration curve was established with known concentrations of a solution of HSPC in chloroform.

The amount of cisplatin and mifepristone in the liposomes was measured immediately after being encapsulated. The liposomes were evaluated with HPLC to confirm encapsulation and measure the concentration of cisplatin and mifepristone. The analysis of cisplatin was based on the method published by our group [26]. Briefly, an aliquot of liposomes was transferred to a centrifuge tube and mixed with acetonitrile. The solution was centrifuged at 10,000 rpm and 4 °C, and then the supernatant was dried and resuspended in saline solution using nickel chloride (NiCl_2_) as the internal standard. Cisplatin was derivatized with sodium diethyldithiocarbamate (DDTC) and extracted with chloroform. After vigorous mixing followed by centrifugation, the chloroform layer was injected in a Waters Alliance 2695 HPLC system (Waters Corporation, Milford, MA, USA) fitted with a Waters 2489 UV detector and a Symmetry C18 column (Waters Corporation, Milford, MA, USA). The mobile phase consisted of water/methanol/acetonitrile delivered at 1.8 mL/min. Detection was set at 254 nm.

Mifepristone was analyzed based on a previously reported method with promegestone as the internal standard [27]. Briefly, an aliquot of liposomes was transferred to a centrifuge tube, acetonitrile was added, and the solution was centrifuged at 10,000 rpm and 4 °C. The supernatant was dried and resuspended in the mobile phase (water/acetonitrile) and the sample was injected into the HPLC system. The mobile phase was a water/acetonitrile mixture delivered at 0.8 mL/min, and the detection system was set at 302 nm.

The entrapment efficiency (EE) for cisplatin and mifepristone was calculated by the following formulas:

EE (%) = (amount in liposomes/initial amount) × 100

Drug release was tested in vitro by using Franz diffusion cells assembled with a polycarbonate membrane containing 0.05 μm pores (Millipore Corporation, Burlington, MA, USA) at room temperature. An aliquot of L-Cis/MF was placed into the donor cell compartment (1 mL) and tamped down to the polycarbonate membrane. At predetermined intervals (1, 2, 4, 8, 12, 24, 48, 72 and 96 h), the whole receptor phase medium (5 mL) was removed and replaced with an equal volume of fresh medium. The receptor phase was a saline solution for cisplatin and 2% sodium lauryl sulfate for mifepristone. The amount of each drug released was determined by using the HPLC methods described previously, calculating the percentage of free cisplatin or mifepristone by the following equation:

% Release = (W_t_/W_o_) × 100

where W_t_ is the amount in the receptor phase and W_o_ is the initial amount of cisplatin or mifepristone placed in the donor phase from L-Cis/MF.

The chemical stability of the liposomes was evaluated by measuring the concentration of the cisplatin and mifepristone.

### 2.4. Studies in Cervical Cancer Cell Line

The human HeLa (ATCC^®^ CCL-2™) cell line was obtained from ATCC (American Type Culture Collection, Gaithersburg, MD, USA) and was routinely cultivated in Dulbecco’s modified Eagle medium (Thermo Fisher Scientific, Waltham, MA, USA) supplemented with 10% fetal bovine serum at 37 °C in a 5% CO_2_ atmosphere. Cells were harvested with 0.025% trypsin and 1 mM EDTA and cells between the third and seventh passage were used for in-vitro experiments. For the treatments, the cells were counted using the TC20 Automated Cell Counter (Bio-Rad Laboratories, Inc., Hercules, CA, USA), and they were seeded in 6-well plates (Costar, MA, USA) at a density of 2 × 10^4^ cells per well. At 24 h, the culture medium was removed and fresh medium with various amounts of L-Cis/MF (0–30 μM), cisplatin (0–30 μM) or mifepristone (0–8.5 µM) was added for 5 days. There were six groups: (1) empty liposomes (L-Control); (2) free cisplatin (Cis); (3) free mifepristone (MF); (4) free cisplatin/mifepristone (Cis/MF); (5) cisplatin-loaded liposomes (L-Cis); (6) cisplatin/mifepristone-loaded liposomes (L-Cis/MF).

At the end of the exposure period, the effect on cell proliferation was evaluated with the XTT assay (sodium 3′-[1-(phenylamino-carbonyl)-3,4-tetrazolium]-bis) (Roche Molecular Biochemicals, Mannheim, Germany). The assay is based on the cleavage of the yellow tetrazolium salt XTT by metabolically active cells to form an orange formazan dye. Briefly, cells were seeded in 6-well plates (Corning, Glendale, AZ, USA) at the aforementioned conditions. At the end of the treatment, XTT was added to each well (final concentration: 0.3 mg/mL) followed by a 4-h incubation under culture conditions. The absorbance of the samples was measured spectrophotometrically at 492 nm on a microtiter plate reader (Multiskan MCC Thermo Electron Corp, Vantaa, Finland). All growth inhibition assays were performed in triplicate and in at least three independent experiments for each assay.

For the cell cycle analysis, the cells (2 × 10^5^) were seeded in 25 cm^2^ plates and synchronized in specific medium, which was replaced after 24 h with fresh medium containing the IC_50_ value (6.5 µM) of L-Cis/MF. The same concentration was used for L-Cis. At days 5 and 9 post-exposure, the cultured cells were harvested, washed twice with phosphate-buffered saline, and fixed with 70% (*v*/*v*) ethanol at 4 °C overnight. Subsequently, the ethanol was removed, and the samples were washed with PBS. Cellular DNA was stained with Guava Cell Cycle Reagent for 30 min (Guava Technologies, Hayward, CA, USA). The data were collected and analyzed on a Guava EasyCyte Flow cytometer with GuavaSoft software (Millipore, Hayward, CA, USA). A minimum of 1 × 10^4^ cells were acquired. At least three independent experiments were performed.

The apoptosis was assessed with the Guava Nexin Kit (Guava Technologies, Hayward, CA, USA). Cells were plated and treated at the same dose used in the cell cycle analysis. The Annexin V binding assay was conducted on days 5 and 9 post-exposure. Cultured cells were harvested, washed twice with PBS, and suspended in Guava Nexin Reagent containing Annexin V-phycoerythrin and 7-aminoactinomycin-D (7-AAD). They were then incubated for 20 min at room temperature according to the manufacturer’s instructions (Guava Technologies, Hayward, CA, USA). The evaluation of apoptosis was made by flow cytometry on a Guava EasyCyte system, acquiring 10,000 cells. The analysis of annexin was conducted on GuavaSoft software (Millipore, Hayward, CA, USA). Three independent experiments were performed.

### 2.5. Animals, Tumor Xenografts and Systemic Toxicity

Athymic (nu/nu) female nude mice 6–7 weeks of age were obtained from the National Institute of Nutrition (INNSZ), Cd. México, México. They were housed under pathogen-free conditions with a 12:12 h light/dark cycle and provided food and water ad libitum. All animal procedures were carried out in accordance with NOM-062-ZOO-1999, Ministry of Agriculture, México; and approved by the Ethics Committee (2017) of the National Institute of Cancer (Ref. No. 010/015/IBI-CB/619/10).

After mice were inoculated s.c. in the back with 5 × 10^6^ HeLa cells, tumor growth was monitored with weekly measurements. Tumors were measured in two perpendicular diameters using a caliper and the tumor volume was determined by using the following relation V = π/6 × (large diameter × [small diameter]^2^) [28]. Once the tumor volume reached approximately 150 mm^3^, the animals were randomized into treatments and control groups and the treatments were initiated. The animals were divided into seven groups (*n* = 5): (1) no treatment; (2) empty liposomes (L-Control); (3) free cisplatin (3 mg/kgweek); (4) free mifepristone (1.3 mg/kg/week); (5) free cisplatin/mifepristone (3.0/1.3 mg/kg/week); (6) cisplatin-loaded liposomes (3 mg/kg/week); (7) cisplatin/mifepristone-loaded liposomes (3/1.3 mg/kg/week). All the treatments were administered for three cycles and the drugs were injected i.p., except for free mifepristone, which was administered s.c.

Weekly evaluation was made of the therapeutic efficacy by measuring the tumor volume, and of systemic toxicity by recording animal weight. Changes in the growth rate of tumors were evaluated in a graph of normalized tumor volume vs. days after treatment. Each normalized tumor volume was calculated as V_Ni_ = V_i_/V_0_, where V_Ni_ represents the normalized tumor volume, V_i_ is the volume at the specific day i, and V_0_ is its own initial volume at day 0. At the end of the 8-week experiment, the mice were euthanized under anesthesia (isoflurane/oxygen 3%).

### 2.6. Statistical Analysis

Data are expressed as the mean ± the standard error of the mean (SEM). Differences were examined with one-way analysis of variance (ANOVA) followed by Tukey’s test using SPSS Base 20.0 software (SPSS Inc., Chicago, IL, USA). Statistical significance was set at *p* ≤ 0.05 for all data.

## 3. Results

### 3.1. Preparation and Characterization of Cisplatin/Mifepristone-Loaded Liposomes

The liposomes loaded with cisplatin and mifepristone were successfully attained by the reverse-phase evaporation method. Determinations were made of the particle size, size distribution (PDI) and surface charge (Zeta potential, calculated by DLS) of L-Cis/MF (Table 1). The average liposome size was around 109 nm and the uniform dispersion of particles (PDI: 0.11 ± 0.02) indicated a homogenous size distribution. The negative surface charge evidenced the stability of the system. The co-encapsulation efficiency was 18% for cisplatin and 25% for mifepristone.

**Figure 1:**
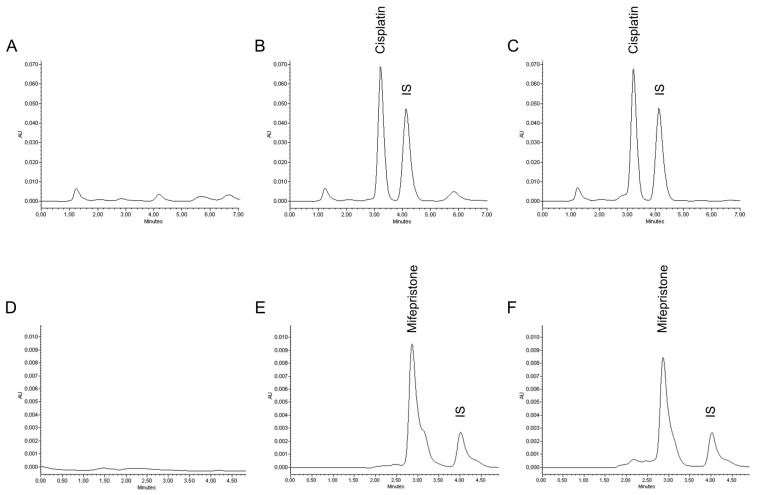
Chromatographic evaluation of the concentration of cisplatin and mifepristone in L-Cis/MF. The images show the peaks for six groups: (**A**) blank liposomes; (**B**) blank liposomes spiked with 10 µg/mL cisplatin and 25 µg/mL NiCl_2_, the internal standard (IS); (**C**) cisplatin drawn from an L-Cis/MF sample and spiked with its IS; (**D**) blank liposomes; (**E**) blank liposomes spiked with 4 µg/mL mifepristone and 4 µg/mL of promegestone (IS); (**F**) mifepristone drawn from a L-Cis/MF sample and spiked with its IS.

The concentration of co-encapsulated cisplatin and mifepristone was evaluated by HPLC. In the typical chromatograms obtained after the extraction of liposomal cisplatin and mifepristone (Figure 1), peaks were observed in the loaded but not blank samples of liposomes. The retention times for cisplatin plus its internal standard (NiCl_2_) were 3.2 min and 4.1 min, respectively, for the blank sample spiked with cisplatin and NiCl_2_, or for the L-Cis/MF formulation. Meanwhile, the retention times for mifepristone plus its internal standard (promegestone) were 2.6 min and 3.8 min, respectively, for the blank sample spiked with mifepristone and promegestone, or for the L-Cis/MF formulation.

**Figure 2:**
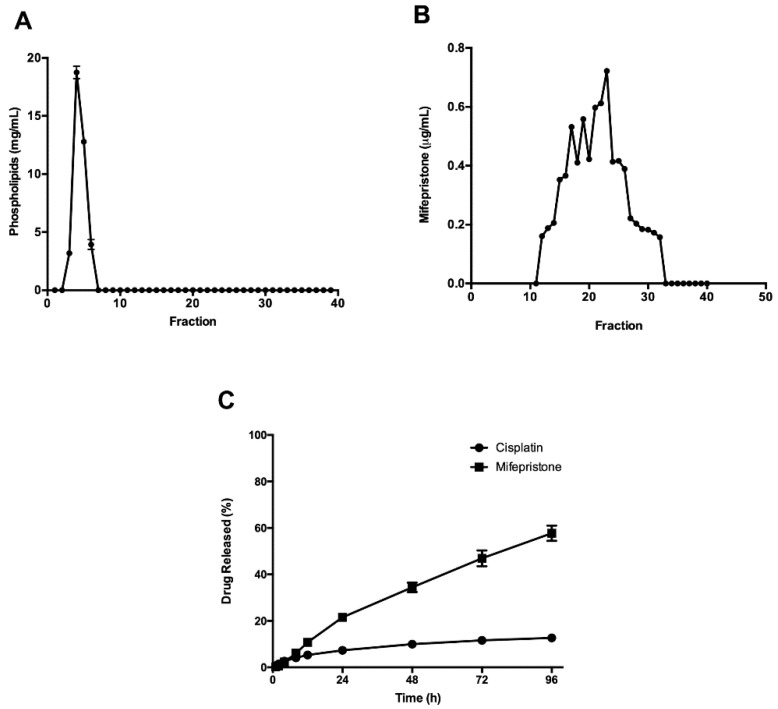
Following the encapsulation of cisplatin and mifepristone in the liposomes, the physicochemical characterization of L-Cis/MF was carried out by measuring: (**A**) the phospholipid concentration in the fractions derived from the liposomes; (**B**) non-encapsulated mifepristone extracted from fractions 11–33; (**C**) the percentage of cisplatin (black circles) and mifepristone (black squares) released from the formulation during 96 h of incubation at room temperature. Data are expressed as the mean ± SD (*n* = 3).

After HPLC, the concentration of co-encapsulated cisplatin/mifepristone was measured in fractions 3–7 and the phospholipid concentration in fractions 1–40 (Figure 2A). Non-encapsulated mifepristone was separated from fractions 11–33 and the concentration was determined (Figure 2B). The results revealed the effectiveness of the liposome purification method.

To analyze the stability of the formulation, drug release profiles were established for cisplatin and mifepristone from L-Cis/MF (Figure 2C). Initially, a slight increase in release was observed for both drugs. After 24 h, however, it was clear that each drug had a different release profile. Cisplatin exhibited a maximum release of approximately 15% at 96 h and mifepristone of about 60%. The concentration of cisplatin and mifepristone in the L-Cis/MF formulation was measured again at ten months, finding that the amount encapsulated cisplatin decreased from 813 ± 103 to 745 ± 6 µg/mL, while mifepristone diminished from 381 ± 19.5 to 365 ± 8 µg/mL, representing a loss of 8% and 4%, respectively.

### 3.2. Post-Treatment Growth Inhibition of HeLa Cells

**Figure 3:**
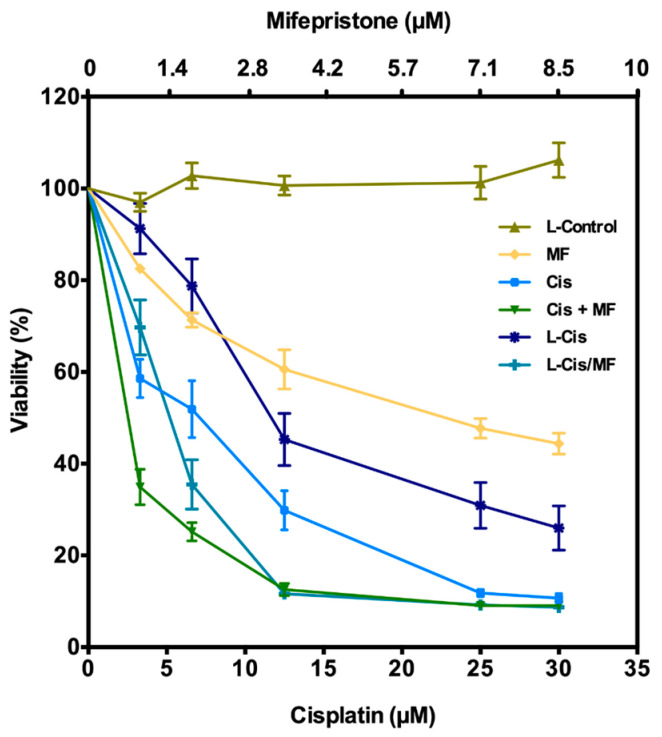
Viability of HeLa cells during the treatments with free cisplatin (Cis), free mifepristone (MF), free cisplatin/mifepristone (Cis/MF), cisplatin-loaded liposomes (L-Cis) and cisplatin/mifepristone-loaded liposomes (L-Cis/MF). The viability assays were repeated in triplicate in at least three independent experiments. Values represent the mean ± SD.

According to the cell viability curve (Figure 3), free cisplatin/mifepristone (Cis/MF) displayed a higher antiproliferative effect than free cisplatin (Cis) and L-Cis. This corroborates our previous study, which found that mifepristone was able to sensitize HeLa cells to cisplatin treatment. Additionally, there was a greater reduction in viability after exposing cells to L-Cis/MF versus L-Cis. Indeed, L-Cis/MF induced a decrease similar to the combination of free cisplatin/mifepristone (Cis/MF). There was a notable cytotoxic effect during the first 5 days of cell exposure cisplatin/mifepristone co-delivered by the L-Cis/MF system and taken in by the cells. The null impact of L-Control (empty liposomes) demonstrates the lack of toxicity of the components of the blank liposomal system.

### 3.3. Cell Cycle and Apoptosis Analysis

**Figure 4:**
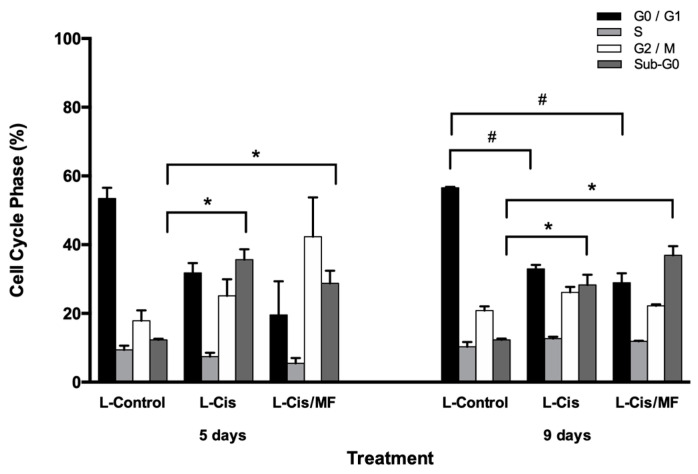
Analysis of the cell cycle for HeLa cells at days 5 and 9 post-treatment in vitro. L-Control (blank liposomes); cisplatin-loaded liposomes (L-Cis) and cisplatin/mifepristone-loaded liposomes (L-Cis/MF). Data are expressed as the mean ± SD of three independent experiments. * and # Significant difference (*p* < 0.05) between L-Control and L-Cis or L-Cis/MF.

Apoptosis and the cell cycle were examined at days 5 and 9 post-treatment of HeLa cells with L-Control (empty liposomes), L-Cis and L-Cis/MF to explore possible mechanisms of the antiproliferative effects of cisplatin/mifepristone-loaded liposomes. Regarding the cell cycle, HeLa cells exposed to empty liposomes displayed an accumulation in the G_0_/G_1_ phases and a decline in population in the S and G_2_/M phases. The drug-loaded liposome treatments changed the distribution of the cell cycle (Figure 4). Five days of exposure to L-Cis/MF generated an increase in G_2_/M and a significant decrease in the G_0_/G_1_ phases. The duration of G_2_/M was shorter for the cells treated with L-Cis versus L-Cis/MF. Both liposome formulations produced a greater cell population in Sub-G_0_ (the sub-diploid phase that indicates apoptotic cells). At day 9, L-Cis and L-Cis/MF showed similar results in the G_2_/M phases as the L-Control. However, the duration of the G_0_/G_1_ phases was still diminished, and the sub-G_0_ phase continued to increase with both liposomal systems, though to a greater extent with L-Cis/MF.

**Figure 5:**
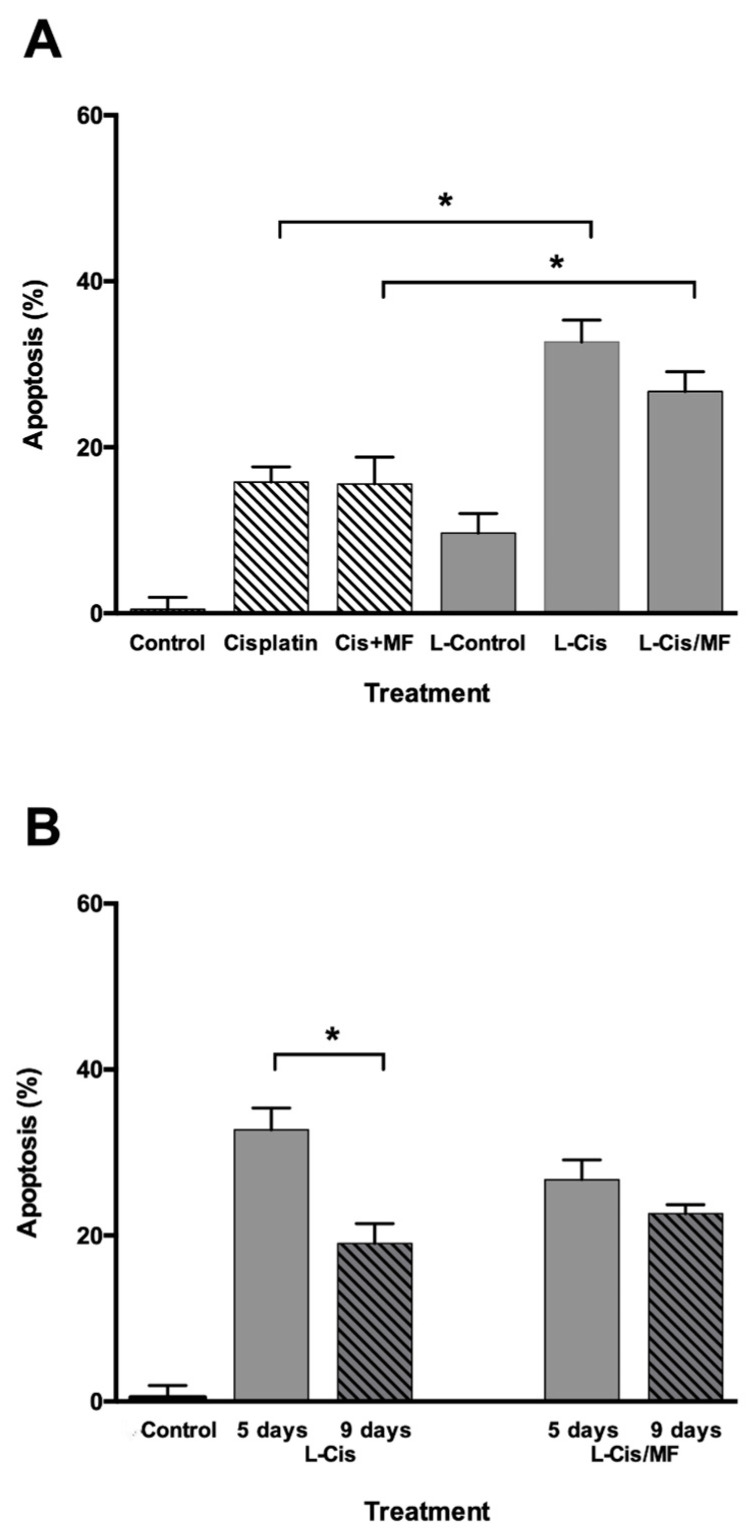
Analysis of apoptosis by flow cytometry in HeLa cells at day 5 post-treatment: (**A**) free cisplatin, free cisplatin/mifepristone (Cis + MF), cisplatin-loaded liposomes (L-Cis), cisplatin/mifepristone-loaded liposomes (L-Cis/MF), and empty liposomes (L-Control). * *p* < 0.05 conventional vs. liposomal treatment. (**B**) Comparison of apoptosis at day 5 and 9 post-treatment with L-Cis and L-Cis/MF. * *p* < 0.05 day 9 vs. 5. Data are expressed as the mean ± SD of three independent experiments.

To further explore the drug-induced reduction in cell viability, early and late apoptosis was examined with Annexin V and 7-AAD, respectively, using flow cytometry (Figure 5). In relation to the administration of L-Control, the conventional treatments (with free cisplatin and cisplatin/mifepristone) increased apoptosis by approximately 18% and the liposome treatments (L-Cis and L-Cis/MF) by about 35%. Hence, there was a significantly higher level of apoptosis triggered by the drug-loaded liposomes versus the free drugs.

The level of apoptosis found at day 5 was compared to that at day 9 post-treatment in each group. At the latter time, the level of apoptotic cells produced by L-Cis was significantly lower, while that generated by L-Cis/MF did not significantly change, suggesting a greater apoptotic effect by the L-Cis/MF system.

### 3.4. Tumor Growth Inhibition in Xenografts

**Figure 6:**
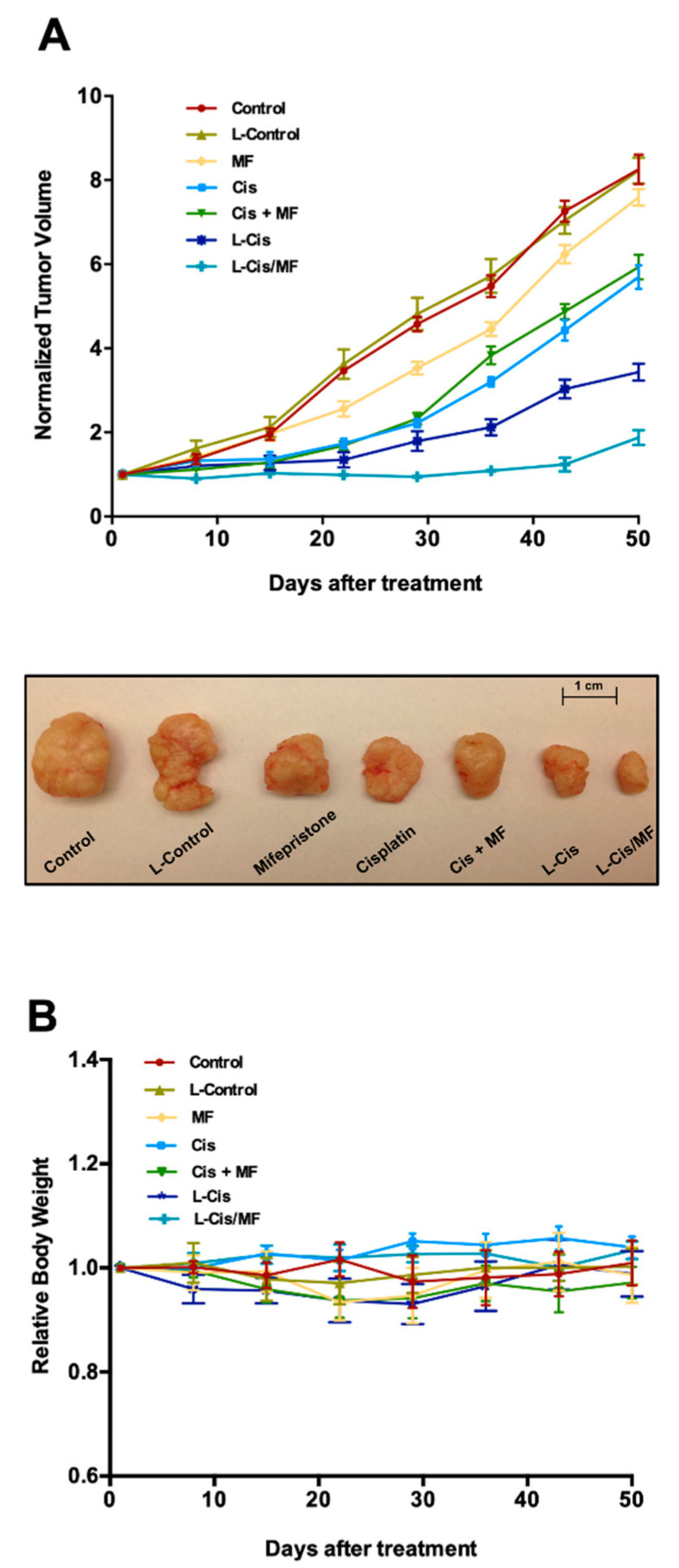
(**A**) Efficacy of the distinct treatments on mice with HeLa cell xenografts: non-encapsulated drugs (cisplatin, mifepristone and the combination of Cis/MF), drug-loaded liposomes (L-Cis and L-Cis/MF), and the corresponding controls: no treatment (control) and empty liposomes (L-Control). HeLa cells were implanted s.c. in the flank of nude mice. The treatments were initiated when the tumors reached 150 mm^3^ (day 0). (**B**) Final body weight of mice in the different groups. Data are expressed as the mean ± standard error of the mean (SEM) of 5 animals per group.

Therapeutic efficacy was studied in vivo using a mouse model with a xenotransplant of HeLa cells and different 3-week treatments (Figure 6). The drug-loaded liposomes (L-Cis and L-Cis/MF) gave the best results. L-Cis/MF produced a significant decrease in tumor growth compared to L-Cis, Cis, MF and Cis/MF. After 50 days of initiating the 3-week treatments, L-Cis/MF proved capable of controlling tumor growth, evidenced by a final tumor volume equal to the initial volume. In relation to the initial tumor volume, an increase was found in all the other groups, being around 8-fold and 6-fold greater with the control and the conventional Cis/MF treatment, respectively (Figure 6A). Regarding possible systemic toxicity, no significant change in animal body weight was observed during the experiment (Figure 6B).

## 4. Discussion

Cervical cancer is a major cause of morbidity and mortality in women worldwide because many patients are diagnosed in the advanced stages. The treatment of choice is cisplatin-based chemotherapy plus radiotherapy. Our group has demonstrated that mifepristone sensitizes cervical cancer cells to cisplatin [12,13].

Due to the lack of specificity of cisplatin, however, only a small quantity of the drug reaches the tumor, which limits the cytotoxic effect on tumors and implies substantial damage to normal tissue. For this and other reasons, cisplatin causes serious adverse effects, including neuropathy and nephrotoxicity, and often leads to drug resistance by tumors [29]. All these circumstances impede the success of the treatment. Incorporation of drugs into liposomes reduces various drawbacks and side effects frequently characteristic of such drugs. Several studies show that PEG-coated liposomes-mediated drug delivery increases the in-vivo circulation, improving plasma half-life, bioavailability and biodistribution of drugs encapsulated, maintaining controlled drug release and enhancing their capability to accumulate in tumors via permeability and retention (EPR) effect. One of the advantages of these nanoparticles is that they do not accumulate in healthy tissue. For example, the liposome exceeds the size cutoff for kidney clearance; therefore, it evades the nephrotoxicity of cisplatin. Canta et al., 2011, showed that lipoplatin, a liposomal formulation of cisplatin, reduced the systemic toxicity of cisplatin; and this formulation was markedly less nephrotoxic and induced less neurotoxicity than conventional cisplatin [30]. There are data that show that lipoplatin does not cause damage to the proximal kidney tubules as does cisplatin for several reasons: cisplatin remains protected by the lipid capsule, and the entrance of lipoplatin particles into the kidney tubule cells is limited; additionally, lipoplatin is released through the kidney with a half-life of 60–117 h compared to 6.5 h for conventional cisplatin, these pharmacokinetic differences could also explain the low renal toxicity [31].

Hence, nanosystems (e.g., liposome nanoparticles) are being elaborated by scientists to encapsulate antineoplastic agents and release them at the desired biological target via targeting ligands directed against endogenous differences between normal and pathologic tissues. Such ligands are systematically selected through their affinity to specific targets and include short peptides, antibodies and antibody fragments, engineered protein domains, and nucleic acid aptamers [32]. This approach aims to improve delivery, reduce toxicity and enhance the antiproliferative effect [33,34]. An analysis was herein made of a liposomal system simultaneously loaded with cisplatin and mifepristone—two drugs with known synergism against several types of cancer including cervical tumors.

Liposomes were prepared with the reverse-phase evaporation method, which has proven to be efficient for the encapsulation of cisplatin [24,35]. Saline solution originally served as the diluent owing to its reported stability [36]. However, the first attempts at encapsulation were not very efficient, giving an average concentration of 615 μg/mL for cisplatin and 175 μg/mL for mifepristone, with a particle size of 184 nm. Consequently, the liposome preparation method was modified by reducing the time of sonication and using distilled water as the diluent. These changes decreased the particle size to 109 nm (with a uniform dispersion of particles: PDI, 0.11) and increased the concentration of encapsulated cisplatin and mifepristone by 60% and 117%, respectively, in the L-Cis/MF system (Table 1).

A particle size of 100–200 nm is reported to favor the passive accumulation of liposomes in tumor tissue due to diminished recognition by the mononuclear phagocyte system (MPS) and reticuloendothelial system (RES) responsible for clearing liposomes from the blood stream [19,37]. Since liposome size influences cellular uptake, tumor permeability and passive targeting, it is a key consideration in any therapeutic application.

The molecular mechanisms of liposome–cell interactions have not been elucidated completely. Initially, mechanisms of interaction that were proposed include: (a) liposome–cell fusion, by considering that liposome membrane fuses with the plasma membrane depending on liposome’s phospholipids; (b) liposome endocytosis by cells; (c) liposome encounters with a low-pH compartment that result in the liposome’s content leak; (d) cell-induced leakage of liposome contents, which is attributed to the interaction of cell surface proteins with the liposome bilayer [38]. More recently, it was proposed that the lipid composition of liposomes determines their surface properties ensuring their accumulation within selected tissue, then the electrostatic potential and surface topology of liposomes must affect the internalization by single cells [39].

The internalization of cationic, neutral and anionic particles into cells is a mechanism of cellular uptake highly dependent on the surface charge of the corresponding particles. In general, cationic liposomes exhibit a preferential uptake in angiogenic tumor vessels of solid tumors through negative charge on the cell surface of angiogenic microvessels. On the other hand, anionic and neutral liposomes revealed clear extravasation from the tumor vasculature, allowing its use as carriers of drugs to the extravascular compartment of tumors [40]. Here, L-Cis/MF has a negative electrical surface potential due to the negatively charged phosphodiester moiety of the phospholipids used in its elaboration (DSPE-mPEG2000 and HSPC). The surface charge of particles can be estimated by the Zeta potential, a parameter that also indicates the stability of suspensions. The forces of electrostatic repulsion between the particles composing a colloidal system determine such stability. Hence, if the surface charge (positive or negative) is increased, the particles will tend to repel each other and remain dispersed in suspension. Particles with Zeta potential greater than +30 mV or lower than −30 mV are generally considered stable [41].

The batches of the present L-Cis/MF system had an average membrane potential of −37 mV, pointing to an acceptable stability. To confirm this supposition, stability was examined 10 months after preparing the liposomal formulation, finding no flocculation or physical changes. Moreover, the amount of encapsulated cisplatin and mifepristone had only decreased by 3.5% and 8%, respectively (Figure 2). Highly charged negative nanoparticles are reported to favor stability because Coulombic repulsion forces arise that can overcome the Van de Waals attractive forces between them and thus prevent aggregation [42,43].

The result of drug release profiles from the L-Cis/MF system show no initial rapid release for either drug. Indeed, both cisplatin and mifepristone were released from the liposomal system in a sustained manner for up to 96 h. However, the two drugs did not exhibit the same pattern of release. Cisplatin was released slowly, reaching a total of almost 15% at 96 h. Contrarily, mifepristone was released more quickly, reaching about 60% at the same time frame. The distinct pattern of release might stem from the difference in hydrophobicity as well as the position of drugs in the liposomal system. Since mifepristone is of lipophilic character [44], it can be integrated into the outer lipid bilayer and consequently is released faster. On the other hand, the hydrophilic character of cisplatin requires it to be located in the internal compartment of the polymeric core [45], and therefore is released at a slower rate.

The amount of cisplatin encapsulated in liposomes is often very limited due to its water insolubility and low lipophilicity [46,47]. The entrapment efficiency of the liposomes for cisplatin in the present system of co-encapsulation was around 18%, similar to the value reported by Hirai et al. and Toro-Cordova et al. [35,47], and for mifepristone was about 25%. To our knowledge, there are no data in the literature on mifepristone-loaded liposomes. In 2003, Hou et al. reported a study in which they used mifepristone as a highly lipophilic model drug to be incorporated into solid lipid nanoparticles (SLNs) [48]; the data showed high drug EE%. However, the drug-release from SLN can be very rapid. In the case of cisplatin-release from SLN, it has been observed to be around 80% after 24 h [49,50] compared to our results where we found that the cisplatin-release was 15% after 96 h. The encapsulated concentration of each drug in the formulation (813 µg/mL for cisplatin and 381 µg/mL for mifepristone) was enough to perform both in vitro and in vivo assays.

Given the achievement in developing liposomes co-encapsulating cisplatin and mifepristone, in vitro and in vivo assays of L-Cis/MF were carried out to assess the synergistic activity of the two drugs. After the HeLa cells were exposed to the treatments in vitro for 5 days, the determination of viability showed a greater decrease in viability afforded by L-Cis/MF than L-Cis, and a similar effect produced by L-Cis/MF and Cis/MF. Hence, the L-Cis/MF system is absorbed by HeLa cells. The in vivo experiment revealed a significantly greater cytotoxicity of L-Cis/Mif compared to all other groups.

It is likely that the effect of drug delivery from the liposomal system is time dependent. The liposomes are probably degraded by lysosome following internalization into cells, causing them to release their therapeutic load. Thus, incubation of the treatments for a longer period may improve anti-tumor efficacy by enhancing interactions with the cellular components. There is evidence of these effects in the cell cycle and apoptosis results.

The progression of the cell cycle was analyzed 5 days after treatment with L-Cis or L-Cis/MF, finding that L-Cis/MF arrests the cancer cells at the G_2_/M phases, while the impact of L-Cis on these phases was not as notable. At 9 days, however, the L-Cis/MF-induced arrest of HeLa cells at G_2_/M was not different from the effect produced by the control group or L-Cis. On the other hand, L-Cis/MF generated a significant increase (versus the controls) in the Sub-G_0_ phase and in apoptosis, and in both cases the elevated level was sustained throughout the 9-day experiment, indicating greater cell death.

After the cytotoxicity study of the liposomal system (both L-Cis and L-Cis/MF), the formulation was evaluated in vivo. For these assays all drugs were injected i.p., and only free mifepristone was injected s.c. It is known that the subcutaneous administration allows the effective administration of certain drugs as hormones included mifepristone. The absorption in general is fast and efficient due to direct access to the blood vessels of the subcutaneous tissue. In the present work, the application of free mifepristone (no-encapsulated) by s.c. was mainly utilized because it is a highly lipophilic drug and its dissolution is poor. To inject mifepristone by i.p., we performed some experiments using a vehicle ethanol:saline solution (10:90) and its dissolution was incomplete. Therefore, we decided to give mifepristone via s.c. to ensure its effectiveness. Additionally, our previous reports suggest that mifepristone administered s.c., improves the efficacy of cisplatin in vivo [12,13]. On the other hand, the intravenous or i.p. application of liposomal formulations are frequently used, this is due to the systemic tolerability after repeated administrations.

Our results demonstrated that the tumor volume was significantly smaller in the animals receiving L-Cis/MF or L-Cis compared to the conventional treatments (Cis/MF or Cis) (Figure 6A); we hypothesized that the liposomal drugs could be reaching tumor tissue at a higher concentration than free drugs resulting in decreased tumor volume. However, further studies of tissue distribution are needed to clearly define the mechanisms responsible for the inhibition of tumor growth after L-Cis/MF administration.

Previous reports from our group have demonstrated the significantly greater drug accumulation in tumor xenografts of the cervix with the systemic administration of a cisplatin liposomal system in comparison to treatment with free cisplatin. This has been attributed to a significantly greater bioavailability and a longer half-life in plasma [26]. However, additional studies of pharmacokinetics are necessary with this new formulation of L-Cis/MF.

The greater cytotoxicity of L-Cis/MF versus L-Cis, observed presently, corroborated the prior finding by our group of the chemosensitizing effect of mifepristone on cisplatin treatment [18,19]. According to other researchers, mifepristone modulates the cytotoxic activity of paclitaxel, cisplatin and doxorubicin—an effect mainly detected in hormone-dependent cancers [17,51,52]. Recently, several mechanisms of mifepristone have been described that are not dependent on hormones [53,54,55,56].

Since the majority of conventional drugs used in chemotherapy are characterized by limited specificity, cytotoxicity and clinical efficacy, an effective liposome delivery system could provide an alternative for improving the safety and efficacy of anti-tumor drugs (e.g., cisplatin). The present liposome formulation exhibited greater cytotoxicity and specificity in the absence of systemic toxicity (judging by stable animal weight). Further research is necessary to examine molecular mechanisms of liposome entry into tumor cells and to carry out bioavailability and biodistribution studies. Our liposome nanosystems capable of co-encapsulating two drugs should certainly be of interest for other types of cancer.

## 5. Conclusions

A liposomal nanosystem was developed to co-encapsulate cisplatin and mifepristone—two drugs that have demonstrated a synergistic effect on several types of cancer cells. The physicochemical characterization of these liposomes indicates efficiency in the encapsulation of both drugs, which led to a stable formulation and sustained drug release. Delivery of cisplatin and mifepristone from the liposomes produced a cytotoxic effect against cervical cancer cells, and a decrease in tumor growth in mice. Evidence was found of cell cycle arrest and apoptosis as mechanisms of cytotoxicity. The liposome nanoparticle carrier system co-loaded with cisplatin and mifepristone clearly improved the effect of chemotherapy. Future studies are necessary to explain the mechanisms related to the biodistribution and pharmacokinetics of liposome nanoparticles that co-encapsulate cisplatin and mifepristone.

## Figures and Tables

**Table 1:** The main physicochemical parameters, measured immediately after the preparation of liposomes, loaded with cisplatin/mifepristone.

Parameter	L-Cis	L-Cis/MF
Particle Size (nm)	132 ± 6.1	109 ± 5.4
Zeta Potential (mV)	−36.1 ± 1.1	−38.7 ± 1.2
Lipids (mg/mL)	29.8 ± 3.4	37.6 ± 0.4
Polydispersity Index (PDI)	0.133 ± 0.03	0.11 ± 0.02
Cisplatin (µg/mL)	989.7 ± 82.9	813 ± 103
Mifepristone (µg/mL)	-	381.2 ± 19.5

Data are expressed as the mean ± standard deviation (SD) (*n* = 3). L-Cis, cisplatin-loaded liposomes; L-Cis/MF, cisplatin/mifepristone-loaded liposomes.
